# Predicting the Health Status of an Unmanned Aerial Vehicles Data-Link System Based on a Bayesian Network

**DOI:** 10.3390/s18113916

**Published:** 2018-11-13

**Authors:** Xiaohong Wang, Hongzhou Guo, Jingbin Wang, Lizhi Wang

**Affiliations:** 1 School of Reliability and Systems Engineering, Beihang University, Beijing 100191, China; wxhong@buaa.edu.cn (X.W.); zy1714114@buaa.edu.cn (H.G.); 2 AVIC Aeronautical Radio Electronics Research Institute, Shanghai 201100, China; radcliffe12@126.com; 3 Unmanned System Institute, Beihang University, Beijing 100191, China; 4 Key Laboratory of Advanced Technology of Intelligent Unmanned Flight System of Ministry of Industry and Information Technology, Beihang University, Beijing 100191, China

**Keywords:** UAV data-link system, Bayesian networks, health status prediction, networking mode, bit error rate

## Abstract

Unmanned aerial vehicles (UAVs) require data-link system to link ground data terminals to the real-time controls of each UAV. Consequently, the ability to predict the health status of a UAV data-link system is vital for safe and efficient operations. The performance of a UAV data-link system is affected by the health status of both the hardware and UAV data-links. This paper proposes a method for predicting the health state of a UAV data-link system based on a Bayesian network fusion of information about potential hardware device failures and link failures. Our model employs the Bayesian network to describe the information and uncertainty associated with a complex multi-level system. To predict the health status of the UAV data-link, we use the health status information about the root node equipment with various life characteristics along with the health status of the links as affected by the bit error rate. In order to test the validity of the model, we tested its prediction of the health of a multi-level solar-powered unmanned aerial vehicle data-link system and the result shows that the method can quantitatively predict the health status of the solar-powered UAV data-link system. The results can provide guidance for improving the reliability of UAV data-link system and lay a foundation for predicting the health status of a UAV data-link system accurately.

## 1. Introduction

Unmanned aerial vehicles (UAVs) are used widely in military and civilian applications because of their low initial cost, high cost-effectiveness over time and ability to operate without casualties [1]. UAVs serve in diverse areas such as exploration, investigation, weather forecasting, pipe network inspection, aerial photography and express delivery services. However, unlike manned aircraft, UAVs require data-link system to link ground terminals to the real-time control of each vehicle. The condition of a UAV data-link system determines whether the UAV can perform its tasks successfully. Therefore, it is particularly important to develop a model for predicting the health status of UAV data-link system.

Because of the complexity and diversity of the tasks carried out by UAVs and the harsh environments in which they may operate, the networking modes of UAV data-links are complex and diverse in order to provide effective control. The failure of a UAV data-link that results in the degradation or failure of performance may also involve accidental failure of hardware and even failure of the link itself. Complex and volatile environments often have an impact on the health of UAV data-link systems. For example, an increase in the bit error rate will reduce the quality of information transmission and affect the health of a UAV data-link. So, we have not found any research on the health status prediction of the UAV data-link system.

Many scholars [2,3,4,5,6,7] have conducted extensive research on health status prediction. For example, Nguyen et al. studied the selection of different degradation models using a large number of health monitoring data [8]. Most of the systems for health status prediction have been modeled based on one of several approaches: the gamma process [9,10,11], Wiener process [12,13], Markov process [14], general generation function [15], Monte Carlo Simulation [16,17,18]. However, these methods can only describe the physical health status caused by performance degradation or accidental failure but the health status of the data-link system is affected by the health status of the hardware, as well as the link health status.

Schumann et al. designed a real-time, on-board system health management (SHM) to the health status of UAV and adopted Bayesian network methods for fault diagnosis [19]. Chonlagarn et al. developed a method to predict the online health status of the UAV system based on hybrid dynamic Bayesian network [20]. Khan et al. proposed a method for predicting the state of health of systems based on artificial intelligence [21] but there is not enough data to build the model. Bayesian networks (BN) as proposed by Pearl [22] provide a reasoning model based on Bayesian theory and graph theory. Graph theory is used to describe a complex system clearly and qualitatively and the probabilistic method is used for quantitative analysis. BNs have obvious advantages for modeling complex systems in areas such as financial risk analysis, wireless sensor networks, system reliability analysis [23] and system health management [24]. Through the use of qualitative network topologies and quantitative conditional probability descriptions, Bayesian networks can clearly represent the inter-component correlation and can integrate information from different sources, including experimental data, historical data and expert experience. In addition, BNs have obvious advantages for describing the multi-level systems [25] used widely in communication quality prediction [26] and the systems involving information interaction [27,28,29]. Many scholars have adopted the Bayesian network to do a lot of research on the system health management of UAV systems. Therefore, we adopted a BN in this research to evaluate and predict the health status of the UAV data-link system.

This paper proposes a UAV data-link health status prediction method based on a BN. This approach combines information about the health status of hardware devices that have different lifetime characteristics with data about links health status as affected by the environment. The degraded health status of the UAV data-link system due to hardware device performance degradation and link failure can be described by this model and provides a unified framework for the health status prediction of the UAV data-link system.

The remainder of this paper is organized as follows: Section 2 provides an overview of BNs, including a summary of the concept, construction and inference algorithms used in Bayesian network models. Section 3 proposes a Bayesian network modeling method for UAV data-links that considers the networking mode and the bit error rate. In Section 4, we present a case study in which we apply our research to a type of solar-powered UAV. Finally, Section 5 provides our conclusions and future work.

## 2. Overview of Bayesian Networks

Bayesian Network is a graphical network model of probabilistic reasoning based on Bayesian theory. It consists of a directed acyclic graph (DAG) and a conditional probability table (CPT). The former determines the qualitative network structure between variables and the latter determines the quantitative relationship between variables.

**Figure 1:**
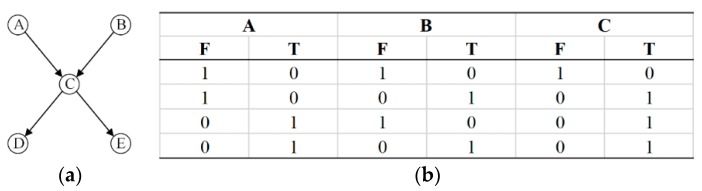
5-node directed acyclic graph and conditional probability table. (**a**) 5-node directed acyclic graph; (**b**) Conditional probability table.

Figure 1a is a 5-node DAG. The attributes of the node variables are arbitrary and can be an abstraction of any problem. The directed edges between nodes represent the interdependencies between nodes and the directed edges are always directed by the parent node to the child nodes. While the variable with no parent node is the root node and the variable with no child node is the leaf node and the rest are the intermediate nodes. The CPT for constructing node C according to the logical relationship is as shown in Figure 1b. As shown in Equation (1), BN probabilistic reasoning is based on the conditional independence assumption that the probability of a child node depends only on the probability of the parent node and is independent of other nodes.

(1) $$p(X_{i}/X_{pi},X_{pai})=p(X_{i}/X_{pi}),$$

where $X_{pi}$ is the parent node of $X_{i}$ and $X_{pai}$ is parent node $X_{pi}$ other children node except $X_{i}$. Applying conditional independence to chain rules enables computation of the joint probability, as follows:

(2) $$p(X_{1},X_{2},\cdots X_{n})=\prod_{1}^{n}p(X_{i}/X_{pi}).$$

Building a BN requires the following steps:

1. Define node variables;
2. Connect the node variables through the directed edges;
3. Establish a CPT for the non-root node.

**Figure 2:**
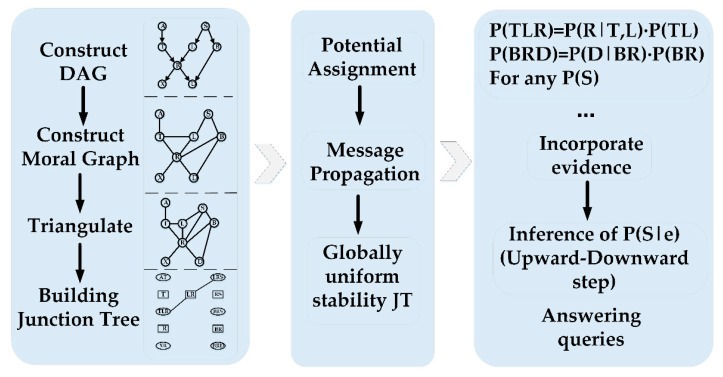
The procedure of Junction Tree (JT) algorithm and build thought of JT. DAG: directed acyclic graph.

After the BN model is constructed, the appropriate inference algorithm is selected for probabilistic reasoning. The Junction Tree (JT) [30] algorithm has been widely used in precision inference algorithms because of its high search efficiency and simple application. The procedure of solution is shown in Figure 2. The first step is to construct the moral map corresponding to the Bayesian network structure, the second step is to triangulate the moral map to obtain the triangulated graph, the third step is to construct the junction tree and the fourth step is to assign parameters to the clusters in the junction tree. The final step is the belief update, which updates the belief in the junction tree using the message propagation algorithm after the evidence added [1].

## 3. Model for Predicting the Health of an Unmanned Aerial Vehicle Data-Link System

**Figure 3:**
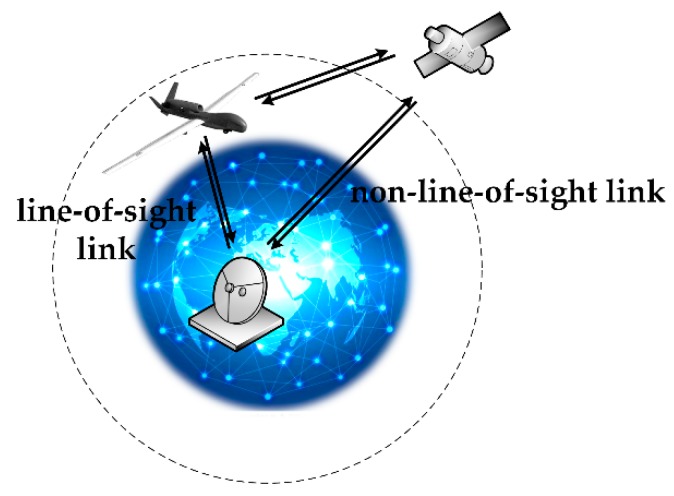
Schematic diagram of unmanned aerial vehicle (UAV) data-link communication mode.

In this section, we present our model for predicting the health of a UAV data-link system based on a Bayesian network. The data-link system for a UAV consists of a part that is airborne and a part that is on the ground. The airborne portion includes the airborne data terminal and antenna. The on-ground portion comprises the ground data terminal and antenna. Both the airborne data terminal and the ground data terminal include a radio frequency receiver, radio frequency transmitter and modem. When the distance between them is relatively close, the UAV and the ground data terminal establish line-of-sight (LOS) wireless communication via the airborne and ground communication units. When the wireless communication line-of-sight link cannot be established because the signal is weakened by long distances or ground obstructions such as buildings, repeater satellites are utilized to establish non-line-of-sight (NLOS) wireless communication. The UAV data-link communication mode is shown in Figure 3.

The link is divided into an uplink and a downlink according to the transmission direction of the information in the link. The ground data terminal transmits a telecontrol command to the UAV through the uplink; the UAV transmits telemetry data, such as the position and attitude of the UAV and other data such as pictures, to the ground data terminal. Both the telecontrol link and the telemetry link must work properly to ensure the UAV data-link system works properly. Therefore, to predict the health status of the UAV data-link system, it is necessary to consider the health status of the telecontrol link and the telemetry link comprehensively. To improve the reliability of UAV data-links, the networking modes of the data-links often adopt a redundant design. UAV data-link networking modes may vary and the information transmission paths may also be different. Because it is often the case that UAVs are used to perform repetitive or dangerous tasks, degradation of the hardware will affect the health status and performance of the UAV data-link. The complex external environment can have a severe impact as well.

### 3.1. Constructing a Bayesian Network Root Node Prediction Model

By analyzing the path of information transmission in the UAV data-link system and the connection relationship between devices, we can use the radio frequency receiver, radio frequency transmitter and modem as the root nodes of the Bayesian network. For ground data terminals and for the UAV and repeater satellites, the radio frequency receiver, radio frequency transmitter and modem can be described with tandem logic as terminals that perform communication functions. Considering that the radio frequency receiver, radio frequency transmitter and modem have no influence on the quality of information transmission, this paper combines these three nodes into communication terminal module nodes for modeling. Under this approach, the sensors are set in each communication terminal module to collect corresponding data and a prediction model of the BN root node is established. Considering that the most important characteristic of the link terminal equipment is the transmission power, the radio frequency device is the key component after the failure mode and mechanism analysis. The power-MOSFET has been adopted for all the airborne terminal equipment in this paper [31]. To establish a predictive model, we combined the degradation model for power-law [32] (considering time), Arrhenius model [33] (considering temperature) and Eyring model [34] (considering electrical stress) with Wiener’s stochastic degradation process [35]. Taking the equipment whose performance degrades from the Wiener process as an example, we introduce the prediction model. Assuming the performance parameter P is a key indicator of product health status and is sensitive to stress S, the parameter then follows the Wiener degradation process as follows [36]:

(3) $$P(t)=d(s)\cdot t+\sigma \cdot B(t)+P_{0},$$

where $P_{\left(t\right)}$ is the product performance at time t and d(s) is the drifting reflecting the performance degradation rate, which is a function of stress and time. In an accelerated model $d(s)=\exp\left[\beta_{0}+\beta \phi \left(s\right)\right]$, where ϕ(s) is a function of stress S; Constant σ is the diffusion coefficient that is irrelevant with respect to environment and time, B(t)~N(0,t) is the standard Brownian motion and $P_{0}$ is the initial value of the parameter.

The degradation amount within the time Δt from the properties of the Wiener process is $\Delta p\sim N(d(s)\Delta t,\sigma^{2}\Delta t)$. L is defined as the failure threshold of performance p and then the time t that performance parameter value first passed through L satisfies the inverse Gaussian distribution. The distribution function is the unhealth state function of the product and the corresponding probability density function is given by:

(4) $$f(t;P_{0},L)=\frac{L-P_{0}}{\sqrt{2\pi \sigma^{2}t^{3}}}\exp\left\{-\frac{{\left[\left(L-P_{0}\right)-d(s)t\right]}^{2}}{2\sigma^{2}t}\right\}.$$

The corresponding health state function is the prediction model of equipment, as follows:

(5) $$R(t)=\Phi \frac{(L-P_{0})-d(s)t}{\sigma \sqrt{t}}-\exp\left\{\frac{2d(s)L}{2\sigma^{2}}\right\}\Phi \frac{(L-P_{0})+d(s)t}{\sigma \sqrt{t}},$$

where Φ(∗) is the cumulative distribution function of the standard normal distribution.

### 3.2. A Bayesian Network Model of an Unmanned Aerial Vehicle Data-Link System Considering the Networking Mode

**Figure 4:**
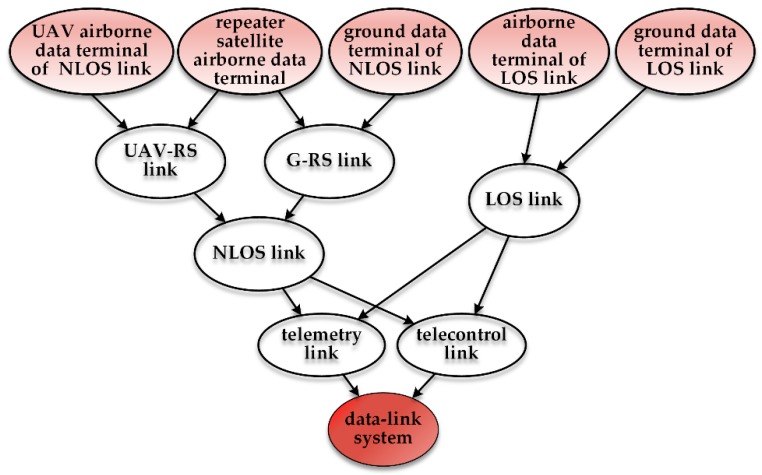
Directed acyclic graph of UAV data-link. LOS: line-of-sight; NLOS: non-line-of-sight; UAV-RS: UAV repeater satellite; G-RS: ground data terminal repeater satellite.

After constructing the BN root node prediction model, we can create the UAV repeater satellite communication (UAV-RS communication) and the ground data terminal repeater satellite communication (G-RS communication) according to the information transfer path and the logical link relationship between the components. Next, we provide a line-of-sight wireless communication link and a non-line-of-sight communication link for the UAV data-link. Both the line-of-sight communication and the non-line-of-sight communication have an impact on the telemetry link and the telecontrol link. Therefore, now we can construct the telecontrol link node and the telemetry link node with the parent node as the line-of-sight communication and the non-line-of-sight communication links. Finally, the leaf node is constructed. The DAG for the UAV data-link system is shown in Figure 4.

In the BN probability prediction model of the UAV-data-link system, $R_{xi}(t)$ $R_{\mathrm{Mj}}(t)$ and $R_{L}(t)$ are used to represent the health state of root node $X_{i}=(i=1,2\cdots p)$, the intermediate node $M_{j}(j=1,2\cdots q)$ and the leaf node L at time t respectively.

For the p root nodes, the probability of health status is solved by the prediction model of each corresponding device and discretized according to the unsupervised equal-width interval method with a time sequence to achieve the state prediction in the future T time; that is, the p×n order health state probability prediction matrix [36]:

(6) $$H_{p\times n}=\left(\begin{array}{ccc} H_{1,1} & \cdots & H_{1,n}\\ \vdots & \ddots & \vdots \\ H_{p,1} & \cdots & H_{p,n} \end{array}\right).\;$$

With a certain time sequence T=(t+ς,t+2ς⋯t+nς). The elements $H_{i,\tau }(i=1,2,\cdots p,\tau =1,2\cdots n)$ represent root node $X_{i}$ to be in the heath state at the τth predicted point. Assigning the prior probability of the root node according to the probability prediction matrix. The probability of the root node $X_{i}$ at time t is $P\left(RX_{i}(t)\right)=H(R_{i}(t)),i=1,2,\cdots ,p$. For the solution of the state probability of intermediate nodes, it is assumed that the parent-node set $F=\left\{R_{1},R_{2},\cdots R_{i}\right\}$ exists for the node $M_{j}$, According to the assumption of independent conditions, the probability prediction of the intermediate nodes at time t can be solved based on:

(7) $$P\left(M_{j}(t)\right)=\sum_{F}P(R_{M_{j}}(t),Rx(t)).$$

Based on the probability of the root node and intermediate node, the predicted probability of the leaf node in health state can be further solved according to:

$$\begin{array}{lll} RL(t) & = & \sum P(Rx1(t),Rx2(t),\cdots ,Rxp(t),RM1(t),RM2(t)\cdots RMq(t),RL(t))\\ & = & \sum_{Pa(L)}P(RL(t)|RPa(L)(t))\cdot \sum_{Pa(Mj)}P(RM1(t)|RPa(M1)(t))\cdots \\ & & \sum_{Pa(Mq)}P(RMq(t)|RPa(Mq)(t))\cdots P(Rx1(t))\cdots P(Rxp(t)) \end{array}$$

where, Pa(∗) is the parent node of the node “(∗)”.

According to the above formula, the JT estimation algorithm traverses the DAG and the state of the system node L can be predicted. By the probability prediction matrix $R_{p\times n}\;=\left(R(t),R(t+\varsigma ),R(t+2\varsigma )\cdots R(t+n\varsigma )\right)$ of the root node, the corresponding prediction sequence of probability at system level will be obtained to achieve continuous prediction of health state.

### 3.3. A Bayesian Network Model of an Unmanned Aerial Vehicle Data-Link System Considering the Bit Error Rate

In addition to the possible degradation of hardware equipment performance and accidental failure, the health status of the UAV data-link system can be affected by the health status of UAV data-links. Such as the bit error rate (BER), packet loss rate, path loss, UAV speed and weather and channel capacity will affect the health status of UAV data-links. It can be expressed as follows:

(8) $$h=f(x_{1},x_{2},x_{3}\cdots x_{n}),$$

where h indicates the health status of UAV data-links, $x_{i}$ is a factor that affects the health status of UAV data-links, such as $x_{1}$ is UAV speed, $x_{2}$ is the weather $x_{3}$ is bit error rate (BER) and so forth. We introduce the bit error rate as a factor affecting the health status of the UAV data-inks in the following paper. In communications, the bit error rate is an important indicator that measures the accuracy of data transmission within a specified time. Often, bit errors are caused by a combination of factors, such as the decay of the signal transmission or pulse interference caused by noise, alternating current, lightning and equipment failure. Since the bit error rate is the number of bit errors divided by the total number of transferred bits during a studied time interval, a probability value can reflect the error (i.e., unhealthy state) of information transmission and therefore it can be used in a BN prediction model as a representation between the nodes that are associated with each of the error transmissions.

**Figure 5:**
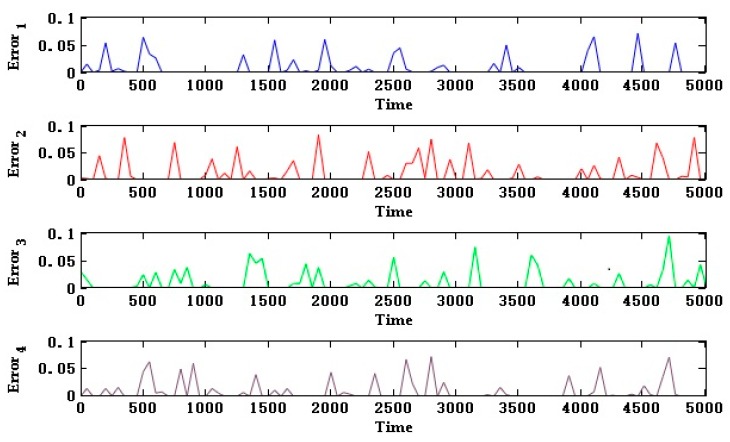
Simulation of bit error rate under different bit stream rates, transmission symbols and signal to noise ratio.

In this way, we can modify the BN model to predict the UAV data-link health state not only considering the networking mode and we can add the bit error rate node to represent the data-link affected by the external environment. The BER value of the newly added root node can be simulated and generated. Each link generates a random bit stream for information transmission by means of the BPSK spread spectrum. The signal is processed by the method of generating the sixteenth order Walsh code and the simulation can be performed. As shown in Figure 5, interfering with the modulated bit stream generates random errors and statistics on the bit error rate, generating a bit error rate value BER*n* for each link, thereby obtaining a probability that the link information transmission is normal: $P_{Error\_n}\;=\;1\;-\;{BER}_{n}$.

The method for predicting the health status of the UAV data-link system proposed in this paper can be summarized as follows:

1. Determining nodes of the UAV data-link system;
2. Construct a DAG of the UAV data-link system and establish the CPT of the non-root nodes;
3. Consider the impact of bit error rate, add the bit error rate nodes and establish the CPT;
4. The JT estimation algorithm is used to solve the joint probability of relevant nodes, to update the conditional probability values of each node and to achieve the deduction of state probability of UAV data-link system nodes.

## 4. Case Study

To test and verify our model, we applied our model to a type of solar-powered UAV data-link system. The solar-powered UAV data-link system consists of two non-line-of-sight links and three line-of-sight links. The B-line-of-sight link (HF band) cannot transmit the task load information of large data such as pictures and images due to the bandwidth and cannot transmit the telemetry information. Therefore, only the telecontrol information can be uploaded in the simplex mode. A-Line-of-sight link (UHF band) and C-line-of-sight link (UHF band) are mutually redundant and the bandwidth of A-line-of-sight link (UHF band) and C-line-of-sight link (UHF band) is sufficient for simultaneous telecontrol and telemetry. Outside the line-of-sight range, the control command is received by the non-line-of-sight links and the long-distance data back-transmission is completed. α-non-line-of-sight link (Ku band) and β-non-line-of-sight link (Ka band) form redundancy. The non-line-of-sight links are relayed by two satellites respectively but the airborne communication terminal can only point to one of them at a certain time and the two non-line-of-sight links share the airborne communication terminal. The normal operation of the UAV is inseparable from the real-time control of the data-link system. The data-link, a subsystem of the unmanned aircraft system that provides for information transmission, has high sensitivity. The airborne communication terminal, repeater satellite’s communication terminal and ground data communication terminal of the unmanned aircraft system are in motion relative to each other at all times. The radio frequency transmitter, radio frequency output power, external interference and the type and gain of the transmitting/receiving antenna determine the maximum acceptable distance for the UAV data-link to establish wireless communication. Data transmission through the UAV data-link will experience channel fading as a result of an increase in the communication distance that goes beyond the range limit, which will have a negative impact on the continuity and stability of signal transmission. This unreliable condition is reflected in the communication quality as the error rate. Based on these considerations, we took the bit error rate and communication distance together as parameters affecting the quality of information transmission to establish the health state prediction model of the UAV data-link system. The factors influencing the quality of each channel of the link were composed of two parts: channel fading caused by increases in the communication distance and the bit error rate caused by random fluctuations.

In our model, the communication interruption rate affected by the communication distance can be described by the Barnett-Vignant formula:

(9) FM=30lgd+10lg(6A⋅B⋅f)−10lg(1−P)−70.

The probability that the information will not be interrupted is:

(10) $$P=1-10^{3\mathrm{lg}d+\mathrm{lg}\left(6A\cdot B\cdot f\right)-7-FM/10},$$

where FM is the fading margin (dB); d is transmission distance (*k*); A is a factor of roughness; B is a factor of climate and environment; and f is the carrier frequency of the channel (GHz).

**Figure 6:**
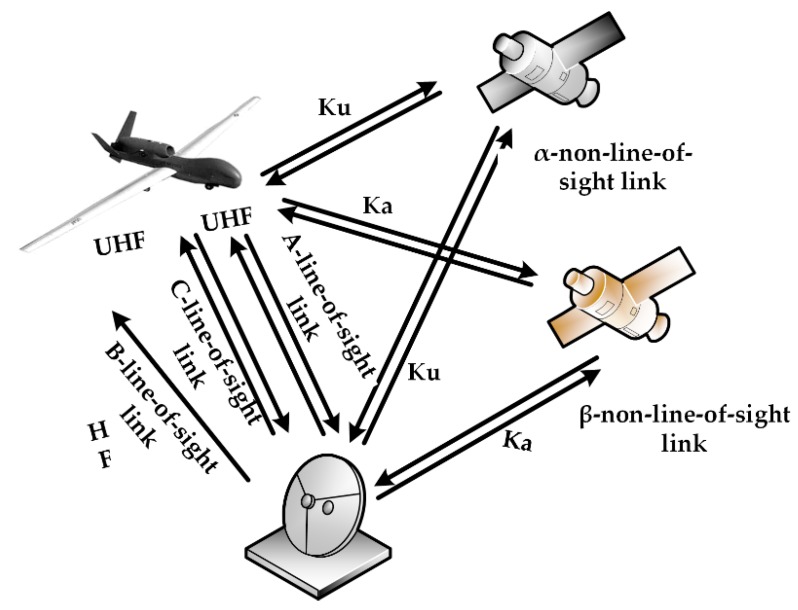
The mode of solar-powered UAV data-link.

Figure 6 shows the mode of the solar-powered UAV data -link system with three line-of-sight links and two non-line-of-sight links. The root node prediction model is shown in Table 1.

**Figure 7:**
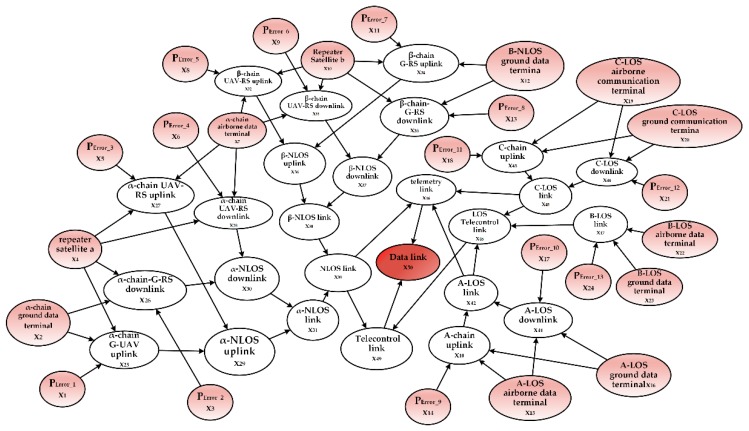
Directed acrylic graph of a solar-powered UAV data-link system.

We built a BN according to the networking mode of the solar-powered UAV data-link system and then we modified the BN by considering the impact of the communication interruption rate and bit error rate. We added 13 root nodes to indicate the factors affecting the health of each channel because of the communication distance and bit error rate. $P_{Error\_1}\sim P_{Error\_13}$ are the BERs of the α-chain non-line-of-sight uplink, α-chain non-line-of-sight downlink, A-chain of the line-of-sight uplink, A-chain of the line-of-sight downlink, C-chain of the line-of-sight uplink, C-chain of the line-of-sight downlink and B-chain of the line-of-sight uplink, respectively. Accordingly, we constructed the BN as show in Figure 7, the leaf node $X_{50}$ respect the health state of solar-powered UAV data-link.

**Figure 8:**
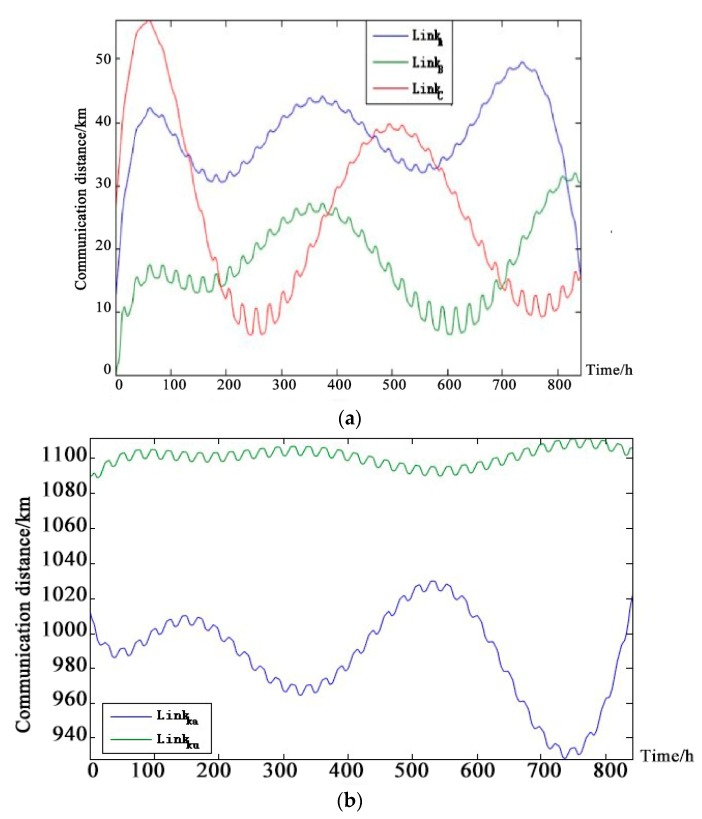
Communication distance distribution curve of each link of solar-powered UAV data-link system. (**a**) Communication distance of UAV-Satellite links; (**b**) Communication distance of ground-satellite links.

Considering the change of communication distance (The solar-powered UAV climbed to an altitude of 8500 m at sunrise and descended to an altitude of 1200 m at night.). Given this diurnal variation of the flying altitudes of the UAV, the communication distances of the line-of-sight chains A, B, C and the non-line-of-sight chain α and β UAV-repeater satellite links are affected by the vertical height and the horizontal distance. The simulation of the communication distance is shown in Figure 8.

**Figure 9:**
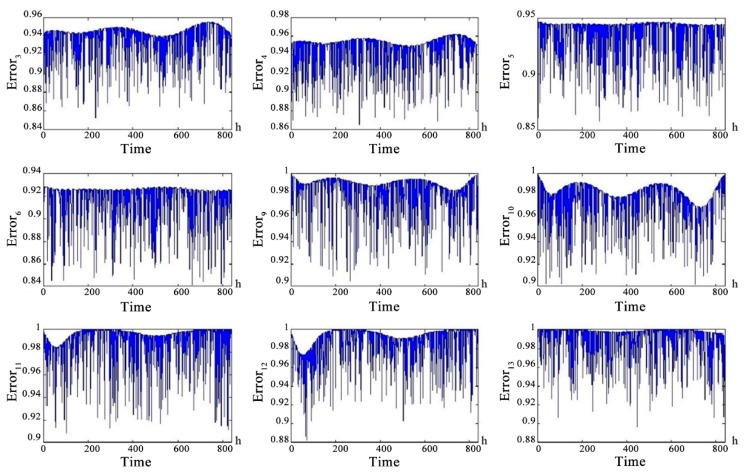
The health status probability of channel with changing communication distance.

According to the communication distance and fading margin (6~10 dB) requirements of each channel, we combined the communication interruption rate simulation curves using the Barnett-Vignant formula and bit error rate to get the health status of each channel. Figure 9 shows the curve $P_{Error\_n}$ (except $P_{Error\_1}$, $P_{Error\_2}$, $P_{Error\_7}$, $P_{Error\_8}$) as a function of time.

**Figure 10:**
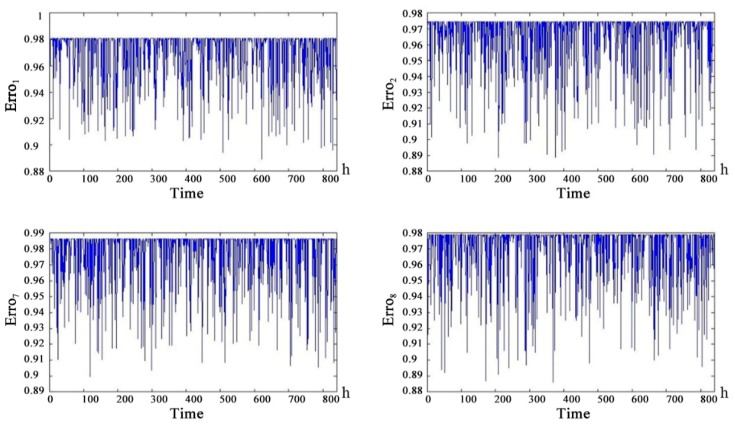
The health status probability of channel with constant communication distance.

For $P_{Error\_1}$ (α-chain ground-satellite uplink), $P_{Error\_2}$ (α-chain ground-satellite downlink) and $P_{Error\_7}$ (β-chain ground-satellite uplink), $P_{Error\_8}$ (β-chain ground-satellite downlink), the distance from the repeater satellite to the ground data terminal can be considered a fixed value because the distance between the repeater satellite and the ground data terminal was much larger than the relative motion distance between the repeater satellite and the ground data terminal. Therefore, the information interruption rate affected by the communication distance was considered a fixed value. We combined the information interruption rate and bit error rate to obtain the curve of $P_{Error\_1}$, $P_{Error\_2}$ and $P_{Error\_7}$ and $P_{Error\_8}$ as shown in Figure 10. The communication distance of the channel is considered to be a fixed value but the health of the channel still produces random fluctuations over time. The health status of the uplink is slightly better than the health status of the downlink.

**Figure 11:**
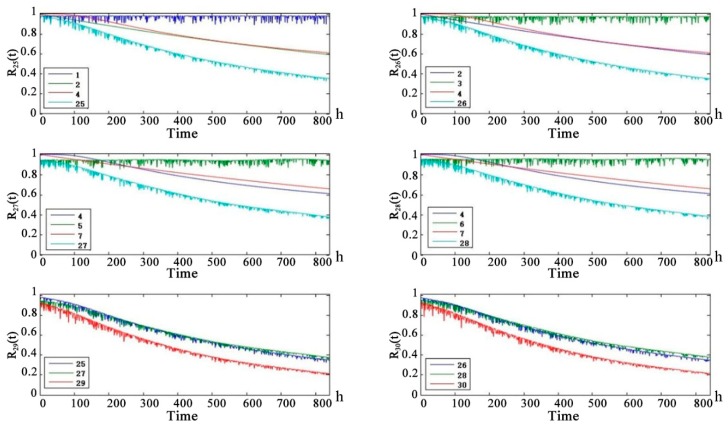
Health state probability prediction curve of Solar-powered UAV data-link intermediate node future 840 h.

**Figure 12:**
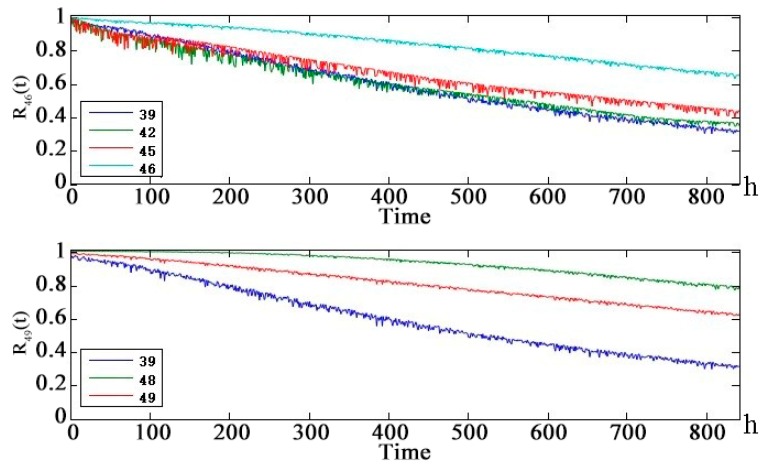
Health state probability prediction curve of Solar-powered UAV data-link intermediate node future 840 h (Continued).

**Figure 13:**
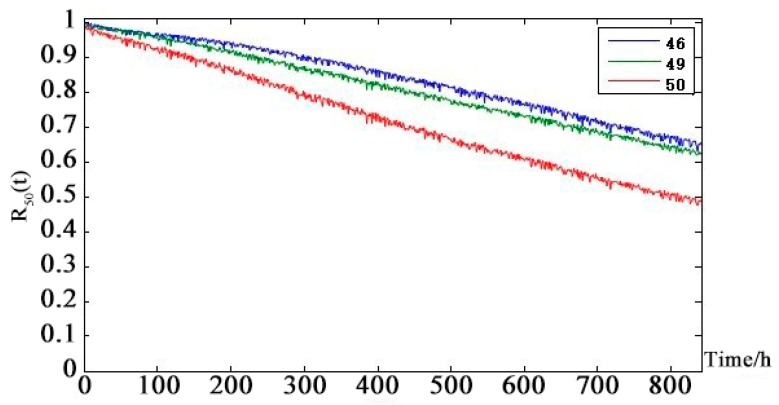
Health state probability prediction curve of Solar-powered UAV data-link leaf node future 840 h.

Figure 11 shows that in the UAV line-of-sight communication link, the health status of the child nodes are worse than that of the parent nodes. The health status of the uplink and downlink is basically the same. α-chain G-RS uplink and downlink medium life are approximately 450 h. α-chain UAV-RS uplink and downlink medium life are approximately 550 h. α-NLOS uplink and downlink medium life are 350 h. Figure 12 shows that the health of the line-of-sight links are better than the health of non-line-of-sight links. Because of the redundancy of links, the telemetry link and telecontrol link did not reach the median life at 860 h. The telemetry link is redundant by 3 links and the telecontrol link is redundant by 2 links, so the health status of the telemetry link is slightly better than the health status of the remote link. Figure 13 shows that the health status of the UAV data-link system is worse than the health status of the telemetry link and the remote link. After 250 h, there is a significant difference. The UAV data-link system reaches the median life about 840 h.

Figure 13 shows that although changes in the position of the solar-powered UAV during the mission changed the communication distance of each channel periodically, the health status of the entire solar-powered UAV data-link system tended to be stable. It can be seen from the data-link system health state prediction curve that both node 46 (telemetry link) and node 49 (telecontrol link) have an impact on the health status of the data-link system node 50 in the next 840 h but the latter is the main reason for the decline of system health status. The weak link is analyzed layer by layer using the prediction curve of the intermediate node: telecontrol → non-line-of-sight link → β-chain non-line-of-sight link → UAV-RS uplink → repeater satellite b. At the same time, it can be seen from the DAG diagram of the solar-powered UAV data-link system that the repeater satellite b node in the β non-line-of-sight link is the parent node of the total of four nodes β-chain non-line-of-sight uplinks and downlinks. So, the repeater satellite b health status has a critical and direct impact on the health of the solar-powered UAV data-link.

## 5. Conclusions and Future Work

In this paper, we proposed a method for predicting the health status of UAV data-link system based on a Bayesian network. This model employs the Bayesian network to describe the information and uncertainty associated with a complex multi-level system. In addition, we proposed an approach considers both hardware equipment degradation and the health status of UAV data-links. This method combines the health status of hardware with different life characteristics and health status of UAV data-links affected by the external environment to predict the health status of the UAV data-link system. We provide a framework to predict the health status of the UAV data-link system, other factors that affect the health status of the UAV data-link system can also be incorporated into this method. In this paper, we describe the hardware health status of different life/performance characteristics and link’s BER value to predict the health status of UAV data-link system with a unified state probability index. Through this approach, we can describe the health status of a UAV data-link system quantitatively and comprehensively.

The case study of a multi-level solar-powered UAV data-link system shows that the model can quantitatively describe the health status of a solar-powered UAV data-link system with hardware degradation failure and link failure affected by communication distance and BER value.

Based on the predicted results, we can understand the health status of the UAV data-link in real time. Based on the predicted results, we can improve the networking mode of the UAV data-link system and provide guidance for the maintenance decision of the UAV data-link system. At the same time, the study laid the foundation for accurately predicting the health of the UAV data-link system.

However, factors such as weather (rain, cloudy), UAV speed, electromagnetic interference and so forth. that have an important impact on the communication quality of the UAV data-link system are not quantified in this paper. In our future work, we will do experiments to get data to verify the indicators of these influencing factors, then more factors can be incorporated into this model, we can accurately predict the health status of the UAV data-link system. At the same time, this method has a high dependence on the prediction model, the more accurate lifetime prediction for UAV data-link system is based on accurate device-level prediction information which imposes higher requirements on information acquisition, processing and analysis from multiple sensors.

## Figures and Tables

**Table 1:** Root node of solar-powered UAV data-link prediction model.

Node	Description of the Prediction Model	Prediction Model and Parameter
α-chain airborne data terminal	degradation model for power MOSFET	$Y=e^{\left(a_{1}+a_{2}\left(\frac{1}{T}-\frac{1}{298}\right)\right)}\cdot \left(t^{a_{3}+a_{4}*\frac{T}{298}}-1\right)+Yo$ $\begin{array}{l} Ea=0.616,A=7.79\times 10^{5},Y_{0}=5,L=8,\sigma =0.08\\ \left(a_{1}=-8.0363,a_{2}=-5529.6,a_{3}=-0.019,a_{4}=-0.8395\right) \end{array}$
α-chain repeater satellite	Wiener process, Arrhenius model	$Ea=0.616,A=7.79\times 10^{5},Y_{0}=5,L=8,\sigma =0.08$
α-chain ground data terminal	exponential distribution	R3(t)=exp[−(t2600)]
A-chain airborne data terminal	degradation model for power MOSFET model	$a_{1}=-7.1342,a_{2}=-5391.4,a_{3}=-0.022,a_{4}=-0.8411$
A-chain ground data terminal	exponential distribution	R5(t)=exp[−(t3500)]
B-chain airborne data terminal	Combined acceleration model	$a_{1}=-7.8220,a_{2}=-5419.2,a_{3}=-0.023,a_{4}=-0.8317$
B-chain ground data terminal	exponential distribution	R7(t)=exp[−(t4500)]
β-chain repeater satellite	Wiener process, Arrhenius model	$Ea=0.64,A=2.115\times 10^{6},Y_{0}=33,L=36.5,\sigma =0.178$
β-chain ground data terminal	exponential distribution	Rs(t)=exp[−(t4500)]
C-chain airborne data terminal	degradation model for power MOSFET	$a_{1}=-8.2334,a_{2}=-5219.5,a_{3}=-0.014,a_{4}=-0.7991$
C-chain ground data terminal	exponential distribution	R9=exp[−(t4000)]
